# Spontaneous, Postpartum Coronary Artery Dissection and Cardiogenic Shock with Extracorporeal Membrane Oxygenation Assisted Recovery in a 30-Year-Old Patient

**DOI:** 10.1155/2016/1048708

**Published:** 2016-03-24

**Authors:** Kathleen E. Knapp, Ricardo A. Weis, Efrain I. Cubillo, Alyssa B. Chapital, Harish Ramakrishna

**Affiliations:** ^1^Department of Anesthesiology, Mayo Clinic, 5777 E. Mayo Boulevard, Phoenix, AZ 85054, USA; ^2^Department of Critical Care Medicine, Mayo Clinic, 5777 E. Mayo Boulevard, Phoenix, AZ 85054, USA

## Abstract

Coronary artery dissection is an infrequent cause of acute coronary syndrome in the general population. There is, however, a greater incidence of spontaneous coronary artery dissection (SCAD) in young women, especially in the peripartum period. However, the majority of cases have favorable outcomes with medical management or percutaneous coronary intervention; coronary artery bypass grafting (CABG) and transplantation are utilized in severe cases. This case is a one of a 30-year-old postpartum female with multivessel SCAD requiring CABG with subsequent biventricular failure and inability to wean from bypass. We believe this is the first reported case in which venoarterial extracorporeal membrane oxygenation (VA-ECMO) was used in the management of biventricular heart failure in a postpartum patient with SCAD.

## 1. Introduction

Acute coronary syndrome (ACS) is typically encountered in patients with a history of coronary atherosclerosis or associated risk factors; spontaneous coronary artery dissection has also been described as a rare but identifiable cause of this disease process. Overall, the incidence of SCAD has been reported to be between 0.28 and 1.1% in the general population. Young women encompass up to 70% of these cases, 30% of which are in the peripartum period [1]. Dissection occurs with separation of arterial layers leading to development of a false lumen resulting in impaired blood flow.

Coronary artery dissection can be categorized as either primary or secondary in nature, with secondary dissections typically being iatrogenic and trauma related [2]. The etiology of primary (spontaneous) dissection is likely related to hormonal, hemodynamic, and autoimmune changes, most of which are encountered frequently in the pregnant patient [1, 3]. As noted by Shahzad et al., the upregulation of eosinophils related to alterations in estrogen and progesterone levels may lead to deposition of eosinophils and subsequent degranulation in the adventitia of coronary arteries propagating dissection [1]. The association with increased coronary artery eosinophilic deposition was also described by Borczuk et al. who studied autopsy reports of peripartum patients who had suffered SCAD [4].

Management options for this conditions range from conservative, medical management to invasive interventions, such as coronary artery bypass grafting (CABG) and even cardiac transplantation [5]. Most reports cite the left anterior descending artery as the initial site of dissection in 60% of cases with recurrent disease being uncommon if initial management was appropriate [1, 3]. A total of 162 case reports were published between 1952 and 2012 on peripartum spontaneous coronary artery dissection (SCAD), the majority of which had favorable outcomes with conservative medical management or percutaneous coronary intervention and stent placement [5]. CABG is reserved for severe cases as is placement of mechanical assist devices as a potential bridge to transplantation [6]. In this case report we describe a young female patient who had severe, recurrent, multivessel spontaneous coronary artery dissection initially managed with cardiac catheterization and drug-eluting stent placement followed by emergent CABG at an outside institution and suffered intraoperative biventricular cardiac failure requiring VA-ECMO support secondary to failure to wean from cardiopulmonary bypass.

## 2. Case Report

A 30-year-old critically ill female with past medical history significant for tobacco use presented to our institution from an outside hospital after failure to wean from cardiopulmonary bypass (CPB) after a two-vessel CABG for management of spontaneous coronary artery dissection. She initially presented nine days after an uncomplicated cesarean delivery to the outside facility with complaints of chest pain. Work-up at that time revealed an ST segment elevation myocardial infarction (STEMI) and the patient was emergently taken to the cardiac catheterization lab for left heart catheterization (LHC). She was found to have dissection of the Right Coronary Artery (RCA) and left anterior descending (LAD) coronary artery. Multiple drug-eluting stents were placed and she was discharged home on aspirin, Plavix, and metoprolol. The patient presented again 7 days later with complaints of recurrent chest pain. Repeat LHC revealed a large dissection from the mid left main (LM) to the ostial circumflex (LCX) arteries with patency of the prior stented vessels. At that time she was taken for emergent coronary artery bypass grafting (CABG) of saphenous vein to LAD and saphenous vein to circumflex.

The intraoperative course was relatively uncomplicated until it was evident that she could not be weaned from CPB despite maximum vasopressor and inotropic support. Intraoperative transesophageal echocardiogram (TEE) demonstrated severe biventricular dysfunction. Subsequently, our institution was consulted to convert the patient to central VA-ECMO. This was completed via the preexisting aortic and a two-stage right atrial cannula placed for CPB. She was maintained on multiple inotropic agents and transported by the team to our facility for a higher level of care. An attempt to transition to peripherally cannulated VA-ECMO via the right femoral artery and vein was unsuccessful secondary to severely limited flow related to grossly visible dissection in the proximal common femoral artery. This defect was immediately addressed with the assistance of the vascular surgeon who reestablished antegrade flow with Fogarty thrombectomy and repaired the arteriotomy site utilizing a vein patch. These interventions resulted in restoration of excellent forward flow. It was thought that this incident was likely iatrogenic given the small nature of the peripheral arteries in combination with their underlying propensity for dissection.

During this operative course, repeat TEE revealed that the patient had a large previously unrecognized atrial septal defect (ASD) in the fossa ovalis which was believed to have intermittent bidirectional flow, measuring 1.5 cm. This was subsequently repaired via an open approach while that patient was on CPB. It was also confirmed by the surgeon using a flow probe that there was interval development of significant thrombosis within the newly placed saphenous vein graft (SVG) to left anterior descending (LAD) artery. It was decided that no operative intervention would be taken to correct this issue and the patient was transported to the ICU in stable condition on VA-ECMO with an open chest through persistent aortic cannula and newly placed R femoral vein cannula.

**Figure 1:**
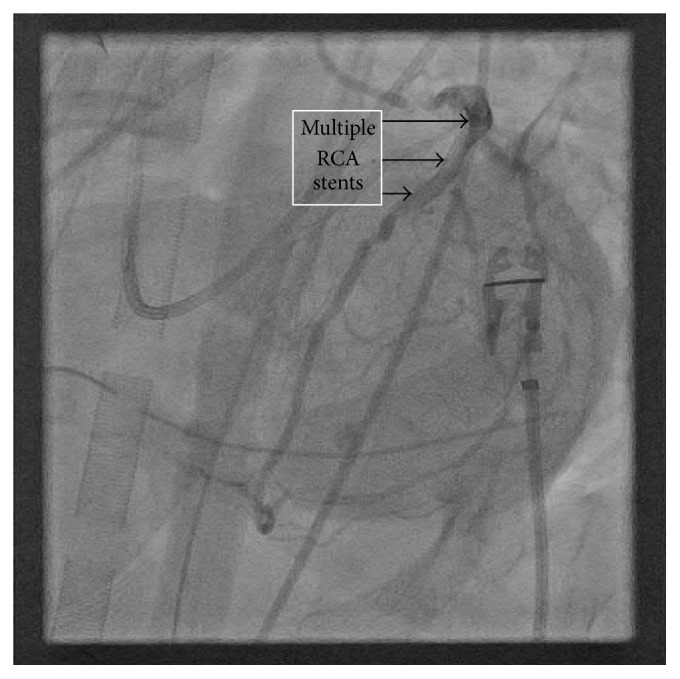
Multiple Right Coronary Artery (RCA) stents as labeled. Visualized during post-CABG cardiac catheterization while on ECMO.

**Figure 2 fig2:**
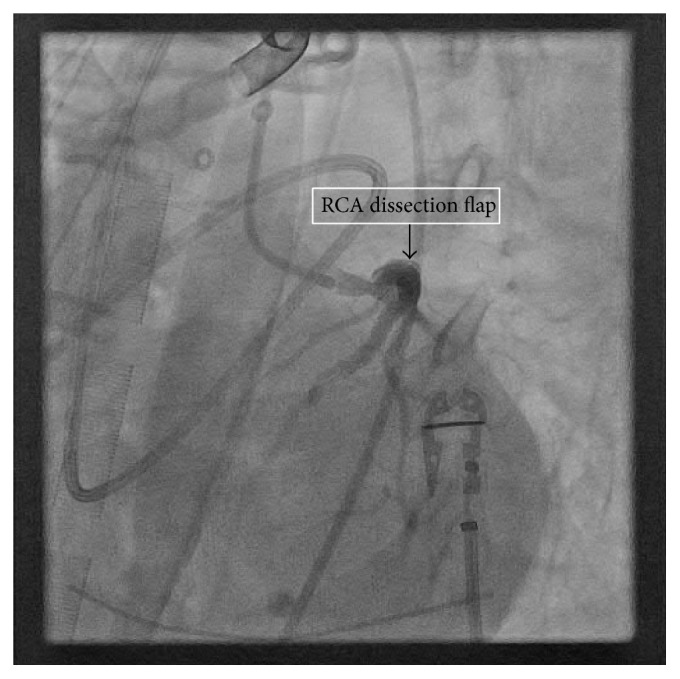
Right Coronary Artery (RCA) dissection flap. Visualized during cardiac catheterization after CABG while on ECMO.

**Figure 3:**
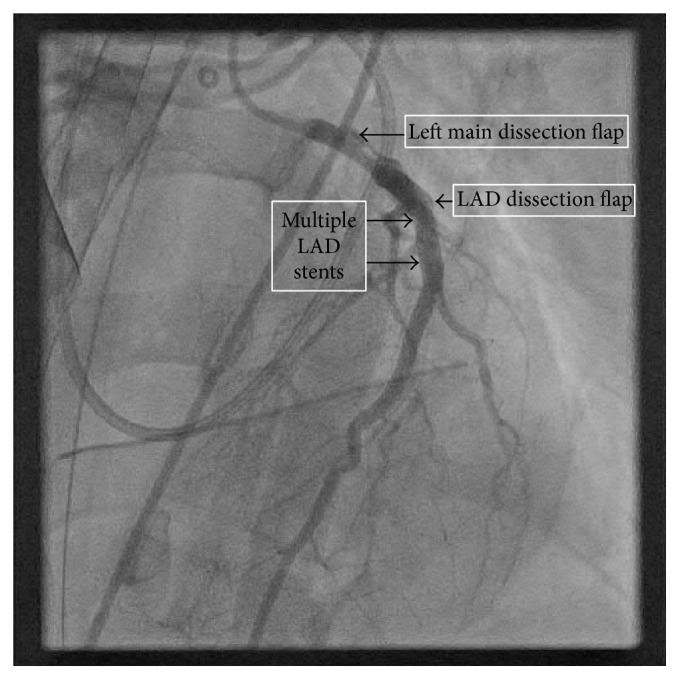
Left main (LM) and left anterior descending (LAD) coronary artery dissection flaps with multiple LAD stents. Visualized during cardiac catheterization after CABG while on ECMO.

Repeat TEE on postoperative day 1 showed persistent postbypass left ventricular dysfunction with an ejection fraction of 25%, global hypokinesis, normal RV size, and mild-to-moderate RV dysfunction. Repeat cardiac catheterization was performed via left common femoral artery to evaluate the possible etiology of her continued dysfunction and new drug-eluting stents (DES) were placed in the native LAD, left main (LM), and the SVG (see Figures 1 [Fig fig2]–3).

She was successfully decannulated four days after VA-ECMO was initiated, at which time TEE revealed improved right ventricular systolic function and LV ejection fraction of 45–50%. Postoperatively, she remained in the ICU with an open chest, on inhaled nitric oxide, epinephrine, and vasopressin. The patient underwent sternal closure on day two status after VA-ECMO decannulation and was ultimately weaned off of inotropes/vasopressors and underwent mechanical ventilation 7 days after VA-ECMO removal. Further work-up into the etiology of her coronary artery dissection failed to reveal any rheumatologic component to her disease. Histopathologic staining was not performed on the operative specimens previously sent to pathology as the rheumatologist did not feel that there was a concern for autoimmune connective tissue disease given that markers including ANA, antiphospholipid antibody, and lupus anticoagulant were negative.

A final transthoracic echocardiogram confirmed improved left ventricular ejection fraction of 48% with mild-to-moderate decrease in the right ventricular systolic function and moderate tricuspid valve regurgitation. The patient was discharged home on hospital day 19 in stable condition. The patient had scheduled follow-up one month after discharge at which time her physical exam showed no overt clinical signs of heart failure. She had self-reported improved exercise tolerance and was instructed to begin phase two cardiac rehab. She was also advised to continue with her current medication regimen of aspirin, Plavix, Coreg, lisinopril, and warfarin. Her case was discussed one last time at the multidisciplinary cardiac transplant selection committee meeting, where she was deemed medically too well to be listed for cardiac transplantation.

## 3. Discussion

Weaning a patient from CPB can be a challenging process especially in the setting of emergent cardiac surgery. The causes for difficulty to wean from CPB are numerous and delineating the exact mechanism may be difficult. The majority of difficult to wean situations can be explained by one of the mechanisms described by Licker et al. in their clinical review of weaning from cardiopulmonary bypass after cardiac surgery. Firstly, hypovolemia in the setting of preserved cardiac output and ventricular function may frequently be the cause of the difficult to wean situation. This should be excluded with TEE evaluation and volume resuscitations. Secondly, according to the authors, it is not uncommon to encounter cardiac structural or dynamic abnormalities as the causative factor. This would include valvular/prosthetic leaks, graft occlusions, and outflow tract obstruction, to name a few. Also described in their review is the importance of vasoplegic syndrome (preserved ventricular function in the setting of low systemic vascular resistance) as a cause of hypotension and inability to separate from the bypass circuit.

Lastly, the cause of difficulty to wean in this case is ventricular dysfunction [7]. Causes of post-CPB ventricular dysfunction are typically multifactorial including surgical tissue trauma, myocardial ischemia-reperfusion injuries, downregulation of beta-adrenergic receptors, coronary embolization (air, atheroma, etc.), activation of inflammatory and coagulation cascades, uncorrected preexisting cardiac disease, and myocardial stunning. There are also patient specific abnormalities that may precipitate a more severe form of dysfunction than initially predicted. With this patient, it is safe to postulate that her multiple unrecognized cardiac structural issues, in combination with factors related to her multiple, prolonged sessions of CPB, were the cause of her severe biventricular dysfunction. Although there are a variety of strategies which may be utilized to successfully separate a patient from CPB (including the use of vasopressors, inotropic agents, and volume resuscitation), in the end, what was beneficial in this case was a mechanical support device in the form of VA-ECMO, more thorough evaluation of her underlying structural defects (ASD repair, repeat LHC with stent placement), and the tincture of time.

ECMO has a variety of uses ranging from the management of patient with acute respiratory distress syndrome to postcardiac surgery patient with significant global hypokinesis. In either instance, the main indications for ECMO support are to maintain right ventricular and/or pulmonary function [8]. Vanzetto et al. evaluated the outcomes of ECMO for life support in low cardiac output states after major coronary surgery and suggested that it is an acceptable modality for temporary circulatory support for the stunned myocardium [6].

While the use of VA-ECMO in this report seems to be unique, there have been many reports which describe the utility of ECMO in caring for peripartum patients. For example, Hansen et al. published a case report in 2012 describing the use of ECMO in a postpartum patient who developed severe, acute mitral regurgitation related to a ruptured posteromedial papillary muscle [9]. Earlier in 2011, Weinberg et al. utilized ECMO in the management of a peripartum patient with massive pulmonary embolism [10]. These examples show the important role that ECMO has played in the management of peripartum patient with significant cardiopulmonary disease. ECMO is being used far more aggressively worldwide in young and otherwise healthy patients with potentially reversible conditions.

Our case report appears to be the first published use of VA-ECMO in a postpartum patient with SCAD requiring emergent on pump CABG who was unable to be weaned from CPB. SCAD has been described in numerous case reports over the years as a potential cause of cardiovascular morbidity and mortality in the peripartum patient. Multiple papers have discussed possible management options for SCAD in the peripartum period; however, currently, there are no guidelines for the management of this specific disease process.
